# Supplementary material for: Work Placement and Job Satisfaction in Long-Term Childhood Cancer Survivors: The Impact of Late Effects
Source: Cancers (Basel). 2022 Aug 18;14(16):3984. doi: 10.3390/cancers14163984 (PMC9406576; doi:10.3390/cancers14163984)
Supplement: Supplementary file 1 [file cancers-14-03984-s001.zip › cancers-1850790-supplementary.pdf]

Supplementary Table S1. Crude and adjusted effects on unemployment, according to the severity of late effects.

	All patients (N=240)						Only employed and unemployed participants (N=187)					
	**Crude effect**			**Adjusted effect**			**Crude effect**			**Adjusted effect**		
	**OR**	**95 % CI**	**p**	**OR**	**95 % CI**	**p**	**OR**	**95 % CI**	**p**	**OR**	**95 % CI**	**p**
**Sex**												
Male	1	[1.00,1.00]	.	1	[1.00,1.00]	.	1	[1.00,1.00]	.	1	[1.00,1.00]	.
Female	0.82	[0.42,1.59]	0.552	0.83	[0.42,1.62]	0.579	0.89	[0.45,1.77]	0.739	0.90	[0.44,1.82]	0.766
**Age at the time of the study (continous)**	1.02	[0.95,1.09]	0.560	1	[0.93,1.08]	0.967	0.96	[0.89,1.03]	0.249	0.94	[0.87,1.01]	0.103
**Age at the first cancer diagnosis (years)**												
18-24	1	[1.00,1.00]	.				1	[1.00,1.00]	.			
25-29	1.01	[0.46,2.20]	0.987				0.61	[0.27,1.39]	0.239			
>=30	1.26	[0.58,2.74]	0.562				0.6	[0.27,1.36]	0.224			
**Late effect intensity**												
No late-effects	1	[1.00,1.00]	.	1	[1.00,1.00]	.	1	[1.00,1.00]	.	1	[1.00,1.00]	.
At least one moderate and none severe	2.03	[0.70,5.92]	0.193	2,06	[0.70,6.04]	0.190	2.05	[0.69,6.12]	0.197	2.30	[0.76,6.97]	0.140
At least one severe	3.22	[1.15,9.00]	0.026	3,21	[1.13,9.12]	**0.029**	3.15	[1.10,9.01]	0.033	3.69	[1.25,10.82]	**0.018**
